# Effects of Soccer Training on Anthropometry, Body Composition, and Physical Fitness during a Soccer Season in Female Elite Young Athletes: A Prospective Cohort Study

**DOI:** 10.3389/fphys.2017.01093

**Published:** 2017-12-22

**Authors:** Melanie Lesinski, Olaf Prieske, Norman Helm, Urs Granacher

**Affiliations:** ^1^Division of Training and Movement Sciences, Research Focus Cognition Sciences, University of Potsdam, Potsdam, Germany; ^2^Olympic Testing and Training Center Brandenburg, Potsdam, Germany

**Keywords:** adolescent athletes, annual training, periodization, training load, strength training

## Abstract

The objectives of this study were to (i) describe soccer training (e.g., volume, types), anthropometry, body composition, and physical fitness and (ii) compute associations between soccer training data and relative changes of anthropometry, body composition, and physical fitness during a soccer season in female elite young athletes. Seasonal training (i.e., day-to-day training volume/types) as well as variations in anthropometry (e.g., body height/mass), body composition (e.g., lean body/fat mass), and physical fitness (e.g., muscle strength/power, speed, balance) were collected from 17 female elite young soccer players (15.3 ± 0.5 years) over the training periods (i.e., preparation, competition, transition) of a soccer season that resulted in the German championship title in under-17 female soccer. Training volume/types, anthropometrics, body composition, and physical fitness significantly varied over a soccer season. During the two preparation periods, higher volumes in resistance and endurance training were performed (2.00 ≤ *d* ≤ 18.15; *p* < 0.05), while higher sprint and tactical training volumes were applied during the two competition periods (2.22 ≤ *d* ≤ 11.18; *p* < 0.05). Body height and lean body mass increased over the season (2.50 ≤ *d* ≤ 3.39; *p* < 0.01). In terms of physical fitness, significant performance improvements were found over the soccer season in measures of balance, endurance, and sport-specific performance (2.52 ≤ *d* ≤ 3.95; *p* < 0.05). In contrast, no statistically significant changes were observed for measures of muscle power/endurance, speed, and change-of-direction speed. Of note, variables of muscle strength (i.e., leg extensors) significantly decreased (*d* = 2.39; *p* < 0.01) over the entire season. Our period-specific sub-analyses revealed significant performance improvements during the first round of the season for measures of muscle power/endurance, and balance (0.89 ≤ *d* ≤ 4.01; *p* < 0.05). Moreover, change-of-direction speed significantly declined after the first round of the season, i.e., transition period (*d* = 2.83; *p* < 0.01). Additionally, significant medium-to-large associations were observed between training and anthropometrics/body composition/physical fitness (−0.541 ≤ *r* ≤ 0.505). Soccer training and/or growth/maturation contributed to significant variations in anthropometry, body composition, and physical fitness outcomes throughout the different training periods over the course of a soccer season in female elite young soccer players. However, changes in components of fitness were inconsistent (e.g., power, speed, strength). Thus, training volume and/or types should be carefully considered in order to develop power-, speed- or strength-related fitness measures more efficiently throughout the soccer season.

## Introduction

In terms of the estimated number of active players, soccer is the most popular sport in the world with more than 270 million participants (Turner and Stewart, 2014). With regards to performance determinants, soccer is an intermittent high-intensity ball game that involves linear sprints, rapid changes-of-directions, jumps and kicks (Bangsbo et al., 2006; Turner and Stewart, 2014). These sport-specific activities require the development of physical fitness during long-term athlete development for successful performance on an elite level (Lloyd and Oliver, 2012; Balyi et al., 2013). Lloyd and Oliver (2012) introduced a physical development model that provides a logical and evidence-based approach to the systematic development of physical fitness in young athletes. This model demonstrates that most, if not all, components of physical fitness are trainable throughout childhood and adolescence. Of note, resistance training is an important means for stimulating athletic development, tolerating the demands of long-term training and competition, and inducing long-term health promoting effects that are robust over time and track into adulthood (Granacher et al., 2016).

In (adolescent) soccer however, seasons are characterized by long competition and short preparation periods. Thus, the time to develop players' physical fitness, to support motor skill acquisition, and to enhance sport performance is limited. Previous studies with young and adult elite soccer players showed that during the season, players are often exposed to prolonged periods of physiological and psychological stress (Michailidis, 2014; Silva et al., 2014; Noon et al., 2015; Rago et al., 2016). For instance, Silva et al. (2014) examined the effects of training and competition during a soccer season in professional soccer players aged 26 years on biochemical stress markers (e.g., creatine kinase activity, myoglobin content) and found significant increases in the level of creatine kinase and myoglobin content during the competition period. Under such conditions, the maintenance or improvement of players' physical fitness depend on appropriate training stimuli that allow the body systems to recover from and adapt to multiple stressors. Thus, to regularly monitoring training data, anthropometry, body composition, and/or physical fitness throughout the season is key for the structured development of performance and the prevention of overuse injuries. In fact, these data are essential to help coaches evaluate their training on a daily basis by tailoring ongoing decision-making processes (Bourdon et al., 2017). In this regard, several studies examined the relationship between measures of training load, anthropomety, body composition, and/or physical fitness in elite adult soccer players over the course of a soccer season (Silva et al., 2011; Mara et al., 2015; Miloski et al., 2016; Jaspers et al., 2017). Findings from these studies indicate significant variations in body composition and physical fitness according to the demands of the respective training period. Further, significant associations were reported between individual match playing time and changes in physical fitness (Silva et al., 2011; Jaspers et al., 2017). Of note, soccer is a complex sport which demands high performance levels in various components of physical fitness (e.g., muscle power, speed, agility) to increase the likelihood for success in competition. For instance, outfield players (e.g., wing-back, central midfield, striker) require well-developed levels of aerobic capacity, speed, agility, maximal strength, and muscle power (Stølen et al., 2005; Bangsbo et al., 2006). Even though these studies provide important information for coaches and practitioners, elite young athletes are different compared to adult athletes and female athletes are different from male athletes in terms of their metabolic (i.e., lower anaerobic capacities) and neuromuscular performance (i.e., lower ability to fully activate muscles) as well as the risk of sustaining injuries (e.g., anterior cruciate ligament injury) (Zauner et al., 1989; Behm et al., 2008; Alentorn-Geli et al., 2009; Faigenbaum et al., 2009; Clemente and Nikolaidis, 2016). Thus, it is not possible to directly translate research findings from (male) elite adult soccer to (female) youth soccer.

To the best of our knowledge, there are only few studies available that examined the effects of a soccer season on anthropometrics, body composition, and physical fitness in elite young soccer players (Williams et al., 2011; Hammami et al., 2013; Di Giminiani and Visca, 2017). Moreover, there is currently no scientific data published on day-to-day training variations, anthropometrics, body composition, and physical fitness at distinct time points over the course of a soccer season in (female) elite young soccer. Thus, we designed a prospective cohort study (i) to describe and evaluate variations in training volume and types, anthropometrics, body composition, and physical fitness over the course of an entire soccer season (e.g., preparation, competition, and transition period) and (ii) to compute respective associations in female elite young soccer players. In accordance with the relevant literature (Silva et al., 2011; Mara et al., 2015; Miloski et al., 2016; Jaspers et al., 2017), we hypothesized that (i) soccer training (and/or growth/maturation) contribute to variations in body composition and physical fitness during the soccer season according to the demands of the respective training period and (ii) the volume of different training types/individual match playing time is significantly associated with relative changes in the respective components of body composition and physical fitness in female young soccer players.

## Materials and methods

### Participants

A team of 19 healthy female elite young soccer players aged 14–16 years at baseline (15.3 ± 0.5 years; Tanner stage 4: *n* = 17 post-pubertal; 2.7 ± 0.4 years post-peak height velocity [PHV]: *n* = 17 post-PHV) participated in this study. The monitored team competed in the first German under-17 soccer division (Junior Bundesliga). Two players who were injured and could therefore not participate in over 75% of training days were excluded from our analyses. Thus, 17 players were finally included in this study. Biological age (i.e., pre-PHV, PHV, post-PHV) was determined according to Mirwald et al. (2002) using time from PHV, based on the Tanner 5-point scale (Marshall and Tanner, 1969). Before entering the study, participants were familiarized with the experimental protocol and potential risks. This study was carried out in accordance with the recommendations of the International Committee of Medical Journal Editors. All subjects and their legal guardians gave written informed consent in accordance with the latest version of the declaration of Helsinki. The protocol was approved by the local ethical commission (University of Potsdam: submission No. 5/2014).

### Experimental procedure

**Figure 1:**
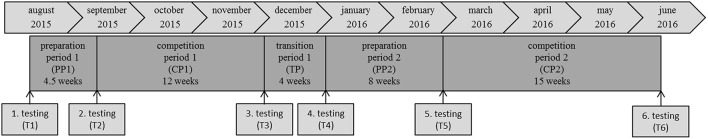
Longitudinal study design. This figure illustrates test sessions over the course of a soccer season.

A prospective longitudinal study design was applied to systematically monitor training and performance data, anthropometrics, and body composition of a female elite youth soccer team during the season 2015/2016. The team completed the season with the German under-17 championship title. All athletes were tested six times over the experimental period (T1-T6; Figure 1) using a large variety of anthropometric, body composition, and physical fitness tests. The fitness test battery included the assessment of muscle strength (i.e., 1-RM of the leg extensors), muscle power (i.e., squat jump [SJ], countermovement jump [CMJ], drop jump [DJ]), muscle endurance (i.e., ventral trunk Bourbon test), speed (i.e., 10-m linear sprint), change-of-direction speed (i.e., T-agility test), dynamic balance (i.e., Y-balance test), endurance (i.e., shuttle run test), and a sport-specific performance test (i.e., kicking velocity). Due to methodological reasons, the tests for muscle strength were conducted only twice over the experimental period (T1 and T6). Prior to physical fitness testing, a standardized warm-up protocol (i.e., 15 min of dynamic stretching, jumping, running and agility/change-of-direction drills) was performed.

### Monitoring of training data

Team coaches tracked day-to-day training data (i.e., volume, types) for each player and each training session over the entire season using an online database (IED database, Institute of Applied Training Science, Leipzig, Germany). Training types were coded as resistance training, sprint training, coordination training, flexibility training, technical training, tactical training, endurance training, and matches (i.e., individual match playing time). Further, specific types in terms of resistance training methods (e.g., hypertrophy training, muscular endurance training) and types (e.g., machine based training, free weights training) were documented. The entire soccer season was divided into five periods: 1. preparation period (PP1; 4.5 weeks; mid-August [T1] until mid-September 2015 [T2]), 1. competition period (CP1; 12 weeks; mid-September [T2] until beginning of December 2015 [T3]), transition period (TP; 4 weeks; beginning of December 2015 [T3] until beginning of January 2016 [T4]), 2. preparation period (PP2; 8 weeks; beginning of January [T4] until end of February 2016 [T5]) and 2. competition period (CP2; 15 weeks; end of February [T5] until mid-June 2016 [T6]) (Figure 1). Before PP1, soccer players returned from a 30-day off-season period in which physical activity was not documented.

### Assessment of anthropometry, body composition, and physical fitness

#### Anthropometry and body composition

At the beginning of each test session (T1-T6), standardized testing protocols were applied for the assessment of standing/sitting body height and leg length (i.e., iliac height). In addition, body composition was analyzed using the InBody720 system (Biospace, Seoul, South Korea). Tests were always conducted at the same time of day.

#### Muscle strength

Maximal leg extensor strength was assessed by means of a 1-RM leg press test on a Cybex Eagle Leg Press (Cybex International, Medway MA, USA). High test-retest reliability was reported previously with an intraclass correlation coefficient (ICC) of 0.997 (Seo et al., 2012). Participants were horizontally positioned on the sledge of the leg-press with hip and knee angles adjusted at 90°. Participants were allowed to stabilize their upper body by holding on to handles attached to the leg-press. Before testing, a warm-up was applied on the leg press using submaximal loads. Subsequently, the 1-RM was determined within five trials using the protocol according to the American College of Sports Medicine (Arena, 2014). Two to four minutes of passive rest were allowed after each trial. All testing procedures were supervised (instructor-to-participant ratio: 1:1). The maximal lifted load (kg) was used for further data analyses.

#### Muscle power

Proxies of muscle power were assessed using SJs, CMJs, and DJs. Jump performance was measured by means of an optoelectric cell system (Optojump, Microgate, Bolzano, Italy). High test-retest reliability was previously reported for the SJ and the CMJ height with an ICC value of 0.97 and 0.98 (Markovic et al., 2004). For the SJ, participants stood quietly in a squatted position (i.e., knees bent 90°), feet shoulder-width apart, and hands akimbo. Jumps were initiated with a concentric upward movement. In terms of the CMJ, participants stood in an erect standing position, feet shoulder-width apart, and hands akimbo. Jumps were initiated with a countermovement which was immediately followed by a concentric upward movement. For the DJ, participants stood in an erect standing position on a 40 cm box, feet shoulder-width apart, and hands akimbo. Participants were asked to step off the box with their dominant leg, drop down to land evenly on both feet on the ground, keep ground contact time short, and jump-off the ground with a double-leg vertical jump at maximal effort. All participants were consistently instructed to jump as high as possible (SJ, CMJ, DJ) and to keep ground contact as short as possible (DJ). Following one test trial, three SJs, CMJs, and DJs were conducted with a rest period of 30 s between the single jump trials and a 1 min rest between the different vertical jump types. The best trial in terms of maximal jump height was taken for the SJ and the CMJ. For the DJ, the best trial in terms of the maximal DJ performance index (i.e., ratio of jump height by ground contact time) was taken for further data analyses.

#### Muscle endurance

The ventral Bourban test was used to assess trunk muscle endurance. The test can be classified as reliable with a coefficient of variation of 14.1% (Tschopp et al., 2001). Participants were in prone bridge position on their elbows and toes. Legs were extended, elbows shoulder-width apart, and forearms rested flat on a fitness mat. While in the bridged position, the lower horizontal reference rod of the alignment device was attached to the participant' s lower back at the level of the iliac crests and was then fixed in this position. After visual inspection of the participant's starting position, athletes were asked to lift their feet alternately for 2–5 cm according to the beat of a metronome (i.e., 1 s per foot). During testing, participants were instructed to remain in contact with the horizontal reference rod for as long as possible. Warnings were given when participants lost touch to the horizontal rod or failed to lift their feet to the beat. The test was terminated when participants failed to remain in contact with the reference rod for the third time. One trial was performed. Test time until failure was manually measured by the tester using a hand-held stop watch and was taken for further analyses.

#### Speed

A 10-m linear sprint test was applied for the assessment of speed. Sprint time was measured using double-light electronic barriers (WITTY; Microgate Srl, Bolzano, Italy). High test-retest reliability was reported for the 10-m sprint test with an ICC of 0.93 (Moir et al., 2004). Participants started the test with one foot 15 cm before the starting line in an erect standing position and were instructed to accelerate as fast as possible. A starting signal was not provided in order to avoid the effect of reaction time. The rest period between the single sprint trials amounted to 3–5 min. The best out of two trials in terms of fastest sprint time was taken for further analyses.

#### Change-of-direction speed

Change-of-direction speed was assessed using the T-agility test (Young et al., 2015). Previously, this test showed high test-retest reliability with an ICC = 0.98 (Pauole et al., 2000). Sprint time was measured using double-light electronic barriers (WITTY; Microgate Srl, Bolzano, Italy). Participants were instructed to run and shuffle as fast as possible following a figure-T course that was set up using four cones. Thus, participants had to continuously change direction throughout the testing procedure. A starting signal was not provided. The rest period between trials was 5 min. Following one test trial, the best out of two trials in terms of fastest sprint time was taken for further data analyses.

#### Dynamic balance

Dynamic balance was assessed using the lower quarter Y-balance test. High test-retest reliability was reported for the Y-balance test in all 3 movement directions with ICC values ranging between 0.89 and 0.93 (Plisky et al., 2006). Before the test started, participants' left and right leg length was assessed in supine lying position by measuring the distance from the anterior superior iliac spine to the most distal aspect of the medial malleolus. Further, participants practiced 3 trials per reach direction on each foot to get familiarized with the testing procedures. All trials were conducted barefooted. The Y-balance test was performed according to the protocol of Plisky et al. (2006). In brief, participants were positioned in single leg stance while reaching as far as possible with the contralateral leg in three different movement directions (i.e., anterior, posteromedial, posterolateral). Participants always started with the right foot placed at the center of the Y-balance test tool (Move2Perform, Evansville, IN, USA) and the left leg reaching three times in anterior direction as far as possible, lightly touching the farthest point possible on the line with the most distal part of the reach foot. Afterwards, the left foot was placed at the center of the grid and the right leg maximally reached in anterior direction. Thereafter, the same test procedure was conducted for the posteromedial and the posterolateral reach direction (positioned 135° from the anterior scale). The examiner manually measured the distance from the scale of the tool. According to Filipa et al. (2010), a composite score was calculated and taken as dependent variable for further data analyses using the following formula: composite score = [(maximum anterior reach distance + maximum posteromedial reach distance + maximum posterolateral reach distance)/(leg length × 3)] × 100.

#### Endurance

Endurance was assessed by means of the 20-m shuttle run test. Test-retest reliability was high with ICCs ranging from 0.91 to 0.94 (Lemmink et al., 2004). The 20-m shuttle run test involves continuous running between two lines which are located 20-m apart according to the timed beep that is delivered by a computer program. Participants stood before starting line and accelerated to the second line on the start signal. Speed at the start was set at 8 km/h. Running speed was controlled through acoustic signals that were delivered by the computer program. Participants continued running between the two lines. When reaching one line, they turned around and ran back to the other line. After about 1 min, an acoustic signal indicated an increase in speed of about 0.5 km/h. This continued each minute (level). If the line was reached before the signal, participants had to wait before running toward the other line. If the line was not reached before the signal, participants received a warning and they had to continue to complete the run toward the line. They then turned around, tried to catch up with the pace within the two subsequent “beeps.” The test was terminated if participants failed to reach the line (within 3 m) for two consecutive acoustic signals. The total distance (m) covered was used for further data analyses.

#### Sport-specific performance

Sport-specific performance was assessed by analyzing kicking velocity during a penalty kick (i.e., ball-goal distance: 11 m) using a standard soccer ball (i.e., FIFA standard size 5) and a Doppler radar gun (Stalker Sport 2, Applied Concepts, Inc./Stalker Radar, Plano, TX, USA). In terms of maximal ball velocity, high test-retest reliability was previously reported (i.e., 0.87 ≤ ICC ≤ 0.93) (Berjan Bacvarevic et al., 2012). Participants were asked to perform three penalty kicks with their dominant and non-dominant leg. Leg dominance was determined according to the lateral preference inventory (Coren, 1993). Athletes were instructed to target the middle of the goal and to act “as forcefully as possible.” Rest between trials was set at 5 min. The best out of three trials for each leg was used for further analyses (i.e., fastest kicking velocity).

### Statistics

Data are reported as means and standard deviations (SD) after normal distribution was confirmed by the Shapiro-Wilk test. Separate analyses of variance (ANOVA) with repeated measures on time (i.e., five levels for the training periods [PP1, CP1, TP, PP2, CP2]; six levels for anthropometry, body composition, and physical fitness test dates [T1-6]) were applied to analyze differences in training data, anthropometry, body composition, and physical fitness. If significant main effects of time were found, a Bonferroni *post-hoc* analysis was conducted. In addition, effect sizes were calculated by converting partial eta-squared to Cohen's d. According to Cohen (1988), effect sizes can be classified as small (0 ≤ *d* < 0.50), medium (0.50 ≤ *d* < 0.80), and large (*d* ≥ 0.80). Finally, associations between training data/individual match playing time and relative changes in anthropometry/body composition/physical fitness (i.e., deltas relative to the duration of the respective training period) were assessed using Pearson's product-moment correlation coefficient. Associations are reported by the correlation coefficient r and level of significance (i.e., in case of multiple correlations we used the Bonferroni correction). Based on the recommendations of Hopkins et al. (2009), values of 0.10 ≤ *r* < 0.30 indicate small, 0.30 ≤ *r* < 0.50 medium, 0.50 ≤ *r* < 0.70 large, 0.70 ≤ *r* < 0.90 very large, 0.90 ≤ *r* < 1.00 nearly perfect, and *r* = 1.00 perfect correlation. The significance level was set at α level < 0.05. All analyses were performed using Statistical Package for Social Sciences (SPSS) version 24.0 (SPSS Inc., Chicago, Illinois, USA).

## Results

### Training volume and types

**Figure 2:**
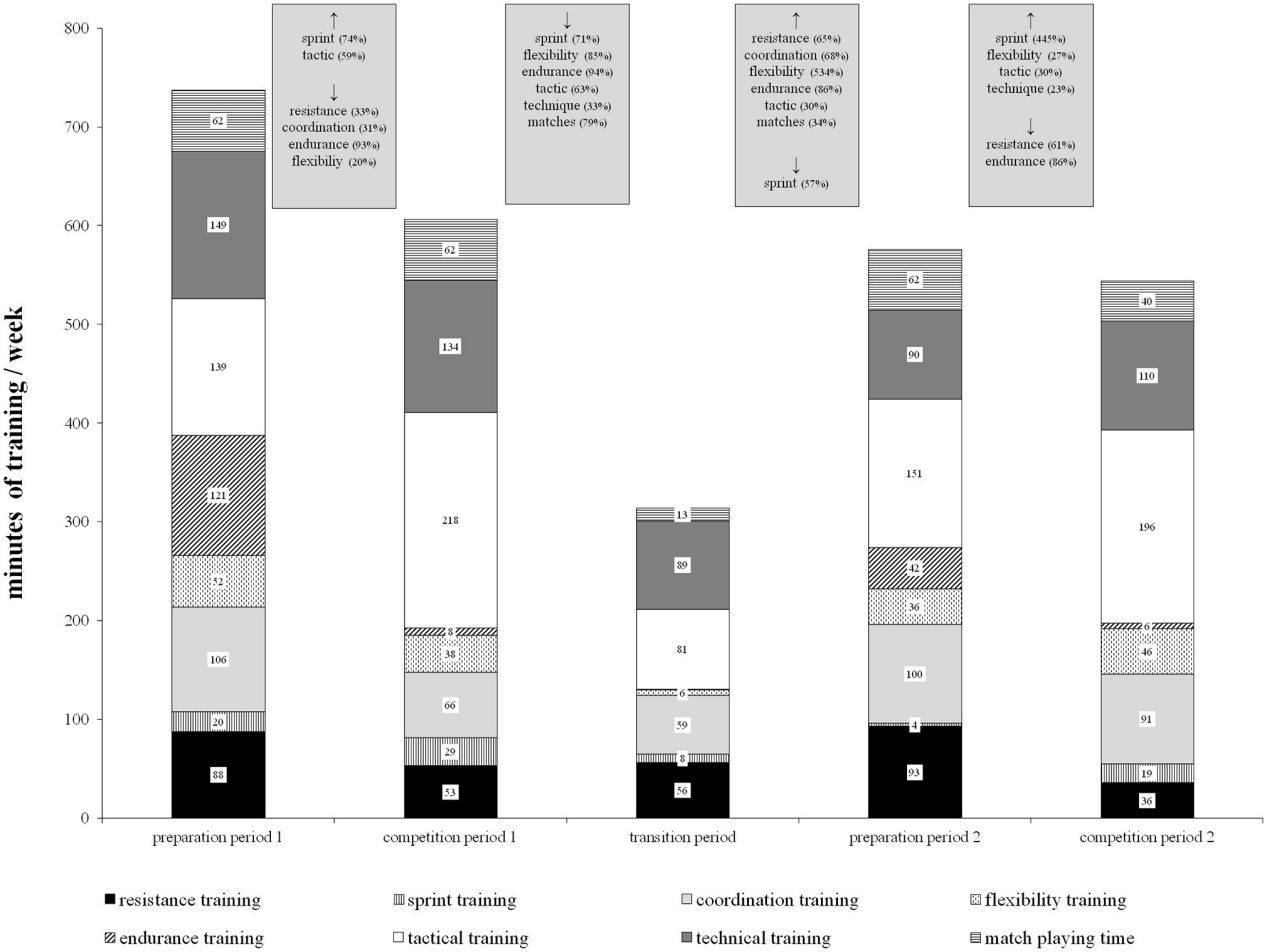
Training volumes classified by training types for each training period in female elite young soccer players. Note that gray boxes denote significant changes. Data are reported as means for the female young soccer team.

Total training volume was 431 ± 21 h (range: 368–458 h) distributed across 339 ± 19 training sessions (range: 284–361 h), and 207 ± 10 (range: 178–220) training days across the soccer season. Training volume for each training period (total and hours per week) is presented in Table 1. Our statistical analyses indicated period-specific training volumes. Due to reduced training sessions per week (i.e., PP1 to CP1: 20% [i.e., 2 sessions/week]; PP2 to CP2: 13% [i.e., 1 session/week]) higher training volumes were found during the preparation periods compared to the respective competition periods (i.e., PP1 to CP1: 15% [i.e., 2 h/week]; PP2 to CP2: 10% [i.e., 1 h/week]). The lowest training volume was found during the transition period. In terms of training types, Figure 2 illustrates the volume of the different training types for each training period. In general, our analyses showed that sport-specific training volume (i.e., technical/tactical training, matches) was significantly larger compared to non-specific training across almost all training periods (52–68% vs. 32–48%), except PP1. Additionally, endurance and resistance training volumes were particularly high during the preparation periods (i.e., PP1, PP2) compared to the competition and transition periods (i.e., CP1, CP2, TP). However, sprint and tactical training volume were particularly high during the competition periods (i.e., CP1, CP2) compared to the preparation and transition periods (i.e., PP1, PP2, TP). The statistical analyses indicated significantly lower training volumes during PP2 compared to PP1 in technique, endurance, flexibility as well as sprint training (Δ30–82%; 2.64 ≤ *d* ≤ 18.96; *p* < 0.01). Further, in CP2 training volume in technique, sprint, and resistance training was significantly lower compared to CP1 (Δ17–35%; 2.18 ≤ *d* ≤ 3.30; *p* < 0.05). In contrast, training volume in flexibility and coordination was significantly higher (Δ22–37%; 2.36 ≤ *d* ≤ 4.69; *p* < 0.001) in CP2 compared to CP1.

**Table 1:** Seasonal changes in training volume in female elite young soccer players.

	**Preparation period I**		**Competition period I**		**Transition period**		**Preparation period II**		**Competition period II**	
	**Total (4.5 weeks)**	**Per week**	**Total (12 weeks)**	**Per week**	**Total (4 weeks)**	**Per week**	**Total (8 weeks)**	**Per week**	**Total (15 weeks)**	**Per week**
Training volume (h)	61 ± 3	13 ± 1	130 ± 9	11 ± 1	22 ± 2	5 ± 0	79 ± 16	10 ± 2	140 ± 10	9 ± 1
Training sessions (n)	51 ± 3	10 ± 1	99 ± 7	8 ± 1	18 ± 1	4 ± 0	64 ± 14	8 ± 2	106 ± 6	7 ± 0
Training days (n)	27 ± 1	5 ± 0	62 ± 4	5 ± 0	12 ± 1	3 ± 0	34 ± 7	4 ± 1	72 ± 4	5 ± 0

*Training sessions = number of sessions (in total and per week) in the respective training period, training days = number of days (in total and per week) in the respective training period*.

More detailed analyses of the different training types according to the applied training methods and types indicated that resistance training comprised muscular endurance training mostly through the application of free weight and stabilization training.

### Anthropometry and body composition

The seasonal variations in anthropometry and body composition are presented in Table 2. Our statistical analyses revealed a significant main effect of time for almost all anthropometric and body composition measures, except for the body mass index. Body height (Δ1%; *d* = 3.39; *p* < 0.001) and total absolute lean body mass (Δ4%; *d* = 2.50; *p* < 0.01) significantly increased over the course of the season (i.e., T1-T6). *Post-hoc* analyses indicated that relative fat mass significantly decreased (Δ8%; *d* = 1.96; *p* < 0.05), while body height, body mass as well as absolute lean leg and lean trunk mass significantly increased (Δ0.3–4%; 1.80 ≤ *d* ≤ 3.36; *p* < 0.05) during CP1. During TP, absolute lean leg mass significantly decreased (Δ3%; 2.80 ≤ *d* ≤ 3.07; *p* < 0.01). During PP2, absolute lean leg mass significantly increased (Δ3%; 3.02 ≤ *d* ≤ 3.03; *p* < 0.01) and relative fat mass significantly decreased (Δ8%; *d* = 1.99; *p* < 0.05). Finally, body height and absolute lean leg mass (right leg) significantly increased (Δ0.3–2%; 2.22 ≤ *d* ≤ 2.25; *p* < 0.01) during CP2.

**Table 2:** Seasonal changes in anthropometry and body composition in female elite young soccer athletes.

	**N**	**Main effect (ES_d_)**	**Start of PP1 (T1)**		**PP1**	**End of PP1 /start of CP1 (T2)**		**CP1**	**End of CP1/start of TP (T3)**		**TP**	**End of TP/start of PP2 (T4)**		**PP2**	**End of PP2/start of CP2 (T5)**		**CP2**	**End of CP2 (T6)**		**Season 2015/2016 (T1 vs. T6)**
			**Mean**	***SD***	**%**	**Mean**	***SD***	**%**	**Mean**	***SD***	**%**	**Mean**	***SD***	**%**	**Mean**	***SD***	**%**	**Mean**	***SD***	**%**
Body height (cm)	14	2.73^***^	165.2	5.7	0.3	165.7	5.7	0.3^*^	166.2	5.6	0.0	166.3	5.6	0.1	166.4	5.6	0.3^**^	166.8	5.8	1.0^***^
Body mass (kg)	15	1.40^***^	56.2	6.7	0.6	56.6	6.0	2.1^*^	57.7	6.2	−0.4	57.5	6.1	0.0	57.5	6.0	1.2	58.2	6.3	3.6
BMI (kg/m^2^)	14	0.67	20.4	1.9	0.0	20.4	1.6	1.3	20.7	1.8	−0.4	20.6	1.8	−0.2	20.6	1.6	0.8	20.7	1.6	1.5
Fat mass (%)	15	1.06^**^	16.4	5.1	2.4	18.5	3.8	−1.4^*^	17.1	3.9	1.1	18.2	3.2	−1.5^*^	16.7	3.0	−0.1	16.6	3.0	1.2
Total lean mass (kg)	15	2.04^***^	26.0	2.8	−1.6	25.5	2.6	4.1	26.6	2.8	−1.8	26.1	2.6	2.2	26.7	2.6	1.5	27.1	2.9	4.4^**^
Right leg lean mass (kg)	15	1.53^***^	7.2	0.9	−0.5	7.1	0.9	2.2^*^	7.3	0.9	−2.5^**^	7.1	0.8	2.9^**^	7.3	0.8	2.1^**^	7.5	0.9	4.0
Left leg lean mass (kg)	15	1.44^***^	7.1	0.8	−0.4	7.1	0.9	2.3^*^	7.3	0.9	−2.7^**^	7.1	0.8	3.0^**^	7.3	0.8	1.5	7.4	0.9	3.7
Trunk lean mass (kg)	15	1.85^***^	19.9	1.9	−2.4	19.4	1.8	4.4^***^	20.2	1.9	−0.5	20.1	1.9	0.1	20.1	1.8	1.3	20.4	2.0	2.8

* *p < 0.05*;

** p < 0.01;

*** *p < 00.1; CP1, 1. competition period; CP2, 2. competiton period; PP1, 1. preparation period; PP2, 2. preparation period; SD, standard deviation; TP, transition period*.

### Physical fitness

**Figure 3:**
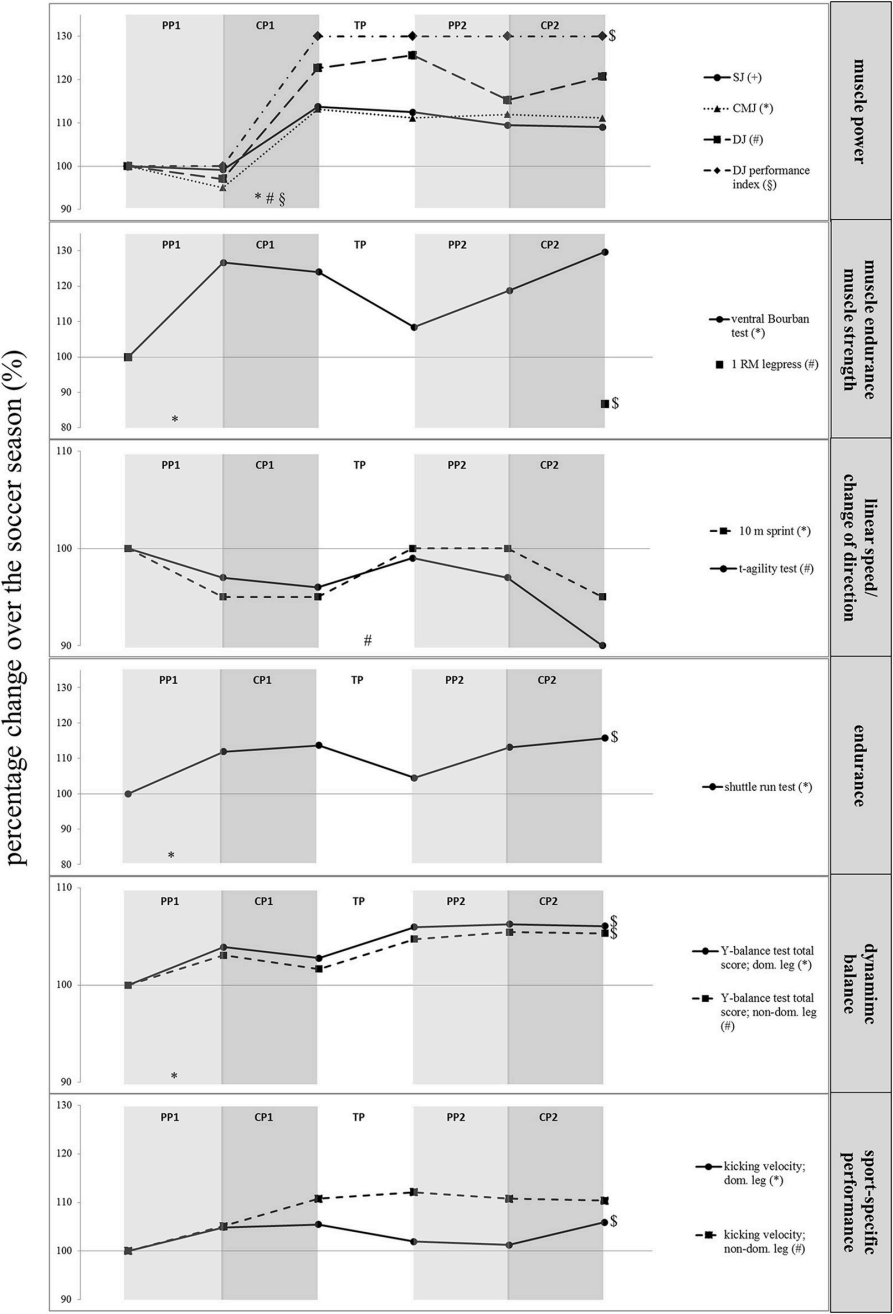
Seasonal changes in physical fitness in female elite young soccer players. For better visual inspection, the vertical axis was not scaled similarly in all charts. Note that improvements in linear sprint and T-agility performances (time) correspond to decreases in percentage changes. CP1, competition period 1 (12 weeks); CP2, competition period 2 (15 weeks); PP1, preparation period 1 (4.5 weeks); PP2, preparation period 2 (8 weeks); TP, transition period (4 weeks); ^*,+,#,§^Significant differences; ^$^significant differences between pre (T1) and post-season (T6).

The seasonal variations in physical fitness are presented in Figure 3. The statistical analyses indicated a significant main effect of time for almost all physical fitness tests, except for the 10 m-sprint. Performances in the y-balance test (Δ5–6%; 2.57 ≤ *d* ≤ 3.95; *p* < 0.05), the DJ performance index (Δ28%; *d* = 3.09; *p* < 0.05), the shuttle run test (Δ16%; *d* = 3.11; *p* < 0.01), as well as kicking velocity of the dominant leg (Δ6%; *d* = 2.52; *p* < 0.01) significantly increased, while maximal leg extensor strength significantly decreased (i.e., 1 RM leg press Δ13%; *d* = 2.39; *p* < 0.01) over the course of the soccer season (i.e., T1 vs. T6). *Post-hoc* tests revealed significant improvements in the ventral Bourban test (Δ27%; *d* = 0.89; *p* < 0.05), the shuttle run test (Δ12%; *d* = 3.28; *p* < 0.01), and the y-balance test of the dominant leg (Δ4%; *d* = 3.18; *p* < 0.01) during PP1 as well as in CMJ height, DJ height, and DJ performance index (Δ19–38%; 2.66 ≤ *d* ≤ 4.01; *p* < 0.01) during CP1. Of note, TP led to significant performance declines in change-of-direction speed (Δ3%; *d* = 2.83; *p* < 0.05). During the second round of the season (i.e., PP2 and CP2), no significant changes in physical fitness were observed.

### Associations between training, match playing time, and changes in anthropometry, body composition, and physical fitness

Significant medium-to-large associations were found between training volumes of different training types/match playing time and the relative changes in anthropometry/body composition (−0.422 ≤ *r* ≤ 0.371) and physical fitness (−0.541 ≤ *r* ≤ 0.505) (Table 3). During CP1 and 2, no significant associations were found between individual match playing time and the relative changes in anthropometry, body composition, and physical fitness (*p* > 0.05).

**Table 3:** Associations between changes in anthropometry, body composition, and physical fitness and the relative volume (in %) of the different training types of the respective training periods in female elite young soccer players.

**Relative changes**	**Resistance training**	**Sprint training**	**Coordination training**	**Flexibility training**	**Endurance training**	**Tactical training**	**Technical training**	**Individual match playing time**
**ANTHROPOMETRY AND BODY COMPOSITION**								
Body mass	−0.143	0.157	−0.197	0.226^*^	0.209	−0.032	−0.197	0.124
Relative fat mass	0.118	−0.010	0.085	−0.073	0.307^**^	−0.356^**^	0.120	−0.163
Absolute lean body mass total	−0.228^*^	0.095	−0.210	0.195	−0.133	0.300^**^	−0.239^*^	0.278^*^
Absolute lean body mass; right leg	−0.201	−0.036	−0.156	0.361^**^	0.078	0.141	−0.380^***^	0.271^*^
Absolute lean body mass; left leg	−0.212	−0.011	−0.225^*^	0.371^**^	0.106	0.158	−0.422^***^	0.304^**^
Absolute lean body mass; trunk	−0.166	0.229^*^	−0.116	−0.002	−0.336^**^	0.354^**^	0.017	0.095
**PHYSICAL FITNESS**								
Countermovement jump height	−0.308^**^	0.293^*^	−0.408^***^	0.054	−0.238^*^	0.401^**^	−0.005	0.226
Squat jump height	−0.341^**^	0.315^**^	−0.408^***^	0.032	−0.079	0.208	0.149	0.148
Drop jump height	−0.070	0.331^**^	−0.272^*^	−0.152	−0.393^**^	0.335^**^	0.230	0.005
Drop jump performance index	−0.028	0.307^*^	−0.246^*^	−0.185	−0.362^**^	0.277^*^	0.262^*^	−0.027
Ventral Bourban test	−0.251^*^	−0.127	−0.311^**^	0.351^**^	0.505^***^	−0.229^*^	−0.310^**^	0.400^***^
1 RM legpress	−0.219	0.239	0.286	−0.264	0.226	−0.333	0.571	0.032
10 m sprint	−0.126	0.075	−0.221	0.056	0.129	−0.096	0.122	0.090
T-agility-test	0.388^**^	0.058	0.267^*^	−0.541^***^	−0.309^**^	−0.059	0.395^***^	−0.296^**^
Shuttle run test	0.122	−0.058	0.089	−0.208	−0.220	0.056	0.276^*^	−0.182
Y-balance test total score, dom, leg	0.279^*^	−0.117	0.158	−0.296^**^	0.256^*^	−0.454^***^	0.292^**^	−0.198
Y-balance test total score, non-dom, leg	0.220	−0.110	0.159	−0.278^*^	0.293^**^	−0.451^***^	0.306^**^	−0.226^*^
Kicking velocitiy, dom, leg	−0.271^*^	0.039	−0.282^*^	0.310^**^	0.325^**^	−0.096	−0.181	0.241^*^
Kicking velocity, non-dom, leg	−0.117	−0.055	−0.130	0.187	0.299^**^	−0.146	−0.120	0.105

* *p < 0.05*,

** *p < 0.01*,

*** *p < 0.001*.

## Discussion

To the authors' knowledge, this is the first study that examined seasonal variations in training data, anthropometry, body composition, and physical fitness in female elite young soccer players. In addition, we computed associations between training/individual match playing time and relative changes in anthropometry, body composition, and physical fitness. The main findings of this study revealed that (i) training volume was significantly higher during PP1/2 compared to TP and CP1/2, (ii) irrespective of the training period, volume of sport-specific training was significantly higher compared to non-specific training, (iii) volume of endurance and resistance training were significantly higher during the preparation periods, while sprint and tactical training volumes were significantly higher during the competition periods, (iv) body height and lean body mass as well as DJ performance index, Y-balance, shuttle run, and kicking performance significantly increased over the course of the soccer season, particularly due to changes in the first round of the season, while maximal leg extensor strength significantly decreased, (v) associations between the volume of different training types/match playing time and relative changes in anthropometry, body composition and physical fitness were unsystematic and reached medium-to-large magnitudes.

### Monitoring training, anthropometry, body composition, and physical fitness

#### Training volume and types

The present study indicates that the annual training cycle of female elite young soccer players significantly varied in terms of volume and type according to the respective training period. This is in accordance with Gamble (2006) who highlighted the training principle of periodization for physical preparation during the season. For instance, we found that weekly training volume decreased from the preparation to the subsequent competition periods. This is in accordance with a previous study from Moreira et al. (2015) who examined pre- and in-season training volumes and intensities in 44 professional Australian football players aged 23 ± 3 years. The authors reported that absolute training volume was significantly higher during the pre-season compared to in-season. Further, training volume relative to period duration significantly decreased from 11 to 13 h/week to 9–10 h/week from the first to the second round of the season. In this regard, Jayanthi et al. (2015) observed that young athletes who participate in more hours of sports per week than number of age in years show an increased risk of sustaining overuse injuries compared to athletes with lower training volumes (odds ratio: 2.07). Thus, the reduction in training volume may decrease the risk of injuries during the competition periods.

Further, our statistical analyses indicated that the training types were specific for the different training periods. More specifically, endurance and resistance training volumes were particularly high during the preparation periods (i.e., PP1, PP2) while sprint and tactical training volumes were particularly high during the competition periods (i.e., CP1, CP2). Moreover, sport-specific training volumes (i.e., technical training, tactical training, matches) were significantly higher compared to non-specific training volumes, but particularly during the competition period (64–68% vs. 32–36%). This is in accordance with the literature (Haff and Haff, 2009; Tønnessen et al., 2014) and indicates higher sport-specific training volumes during the competition periods to better prepare for the upcoming demands of the competition period.

#### Anthropometry and body composition

It has previously been demonstrated that anthropometry and body composition change throughout the course of a soccer season (Mukherjee and Chia, 2010; Hammami et al., 2013; Oyón et al., 2016). For instance, Hammami et al. (2013) and Oyón et al. (2016) evaluated the effects of soccer-training on anthropometric and body composition characteristics (i.e., body height, body mass, relative body fat) in young soccer players. Over the season, Hammami et al. (2013) found significant increases in body height (2%) in elite male young soccer players (15 ± 0.5 years). For female young soccer players (12–15 years), Oyón et al. (2016) reported significant increases in body height (1%), body mass (8%), and relative body fat (2%) over the season. In accordance with the findings of Hammami et al. (2013), we observed significant seasonal increases in body height (1%) only. In contrast to the findings of Oyón et al. (2016), we found no negative changes in body composition (i.e., increase in relative body fat/mass) over the season in the examined sample of female young soccer players. This might be due to differences in training volume in the participants of our study (5–13 h/week) and the study of Oyón et al. (2016) (3 h/week).

Our period-specific analyses indicated no significant changes during PP1 (i.e., T1-T2; 4.5 weeks) in any of the tested anthropometric and body composition data. However, an increase in lean body mass and a decrease in percentage of body fat during CP1 (i.e., T2-T3; 12 weeks) was observed. During TP (i.e., T3-T4; 4 weeks) and at the turn of the year, athletes were granted an active rest in which they conducted non-specific physical activities of their own choice. During this period, training volumes significantly decreased (64–84%) which may have resulted in the observed negative adaptations in body composition that were again compensated during the second round of the season (i.e., PP2, CP2). To the authors' knowledge, there is only one study available (Mukherjee and Chia, 2010) that observed anthropometric and body composition data during the pre-season, the early in-season (i.e., 12 weeks later), and the end mid-season (i.e., again 13 weeks later) in young soccer players. These authors found a significant decrease in relative body fat and a significant increase in lean body mass during the pre-season period in male elite young soccer players aged 18 ± 0.3 years. In contrast, during the competition period Mukherjee and Chia (2010) observed bionegative adaptations (i.e., increase in relative body fat and decrease in lean body mass) in their tested sample. Thus, anthropometry and body composition significantly changed during specific training periods in young soccer players. Additionally, in the present study, small- to-medium sized associations were found between training types and changes in anthropometry/body composition (*r* = −0.422–0.371). Interestingly and in accordance with McManus and Armstrong (2011), female adolescent growth covers a period of 4–4.5 years around PHV until estrogen levels rise and epiphyseal fusion begins which ultimately terminates growth of stature. Even though most of the included female young soccer players were classified as post-PHV, growth can still occur until the age of 17 years (Balyi et al., 2013). All of our study participants were post-PHV with a mean age of 15.3 years. Thus, it is hypothesized that growth and maturation partly contributed to the observed changes in anthropometry and/or body composition. In this regard, Kromeyer-Hauschild et al. (2001) reported similar body height increases (~1%) for German adolescent females aged 15.5 over a one-year period. These changes were similar in magnitude as the ones that were found in this study over the entire soccer season (see Figure S1). Consequently, the reported changes in anthropometry/body composition are multifactorial and can most likely be attributed to training, competition, habitual activity, diet, growth, and/or maturation.

#### Physical fitness

Several health- and skill-related components of physical fitness are essential prerequisites for successful performance in (youth) soccer. In fact, earlier studies identified that higher levels of aerobic endurance (Hoff, 2005; McMillan et al., 2005; Castagna et al., 2006), speed (Murphy et al., 2003; Little and Williams, 2005), agility/change-of-direction speed (Gambetta, 1990; Little and Williams, 2005), strength and power (Wisløff et al., 1998) are important determinants of superior soccer performance (Turner and Stewart, 2014). Several authors observed changes in these physical fitness components over a soccer season (Hammami et al., 2013; Sæther and Aspvik, 2014; Silva et al., 2014; Miloski et al., 2016). Similarly, in the present study we were able to show significant improvements over the season in terms of performances in the Y-balance test, the shuttle run test, the DJ performance index and in kicking velocity (6–28%). However, our findings also indicated that several performance measures maintained (i.e., SJ height, CMJ height, DJ height, 10 m-sprint, change-of-direction speed, ventral Bourban test) or even declined (i.e., maximal leg extensor strength) over the season (T1 vs. T6). This somewhat unexpected finding could partly be explained by the specificity of the training types. Of note, the greatest performance changes occur if training follows the principle of training specificity (Behm and Sale, 1993). In the present study, the training documentation revealed that for instance resistance training predominantly focused on muscular endurance. Plyometric and sprint training constituted only a small part of the overall training volume. Thus, it can be speculated that the volume of specific training stimuli to improve jump, speed, change-of-direction speed and/or leg muscle strength was too low to induce sufficient adaptive processes. From a practical point of view, it appears beneficial to include specific means (e.g., resistance training) in the future that focus on the development of leg muscle strength/power, sprint and change-of-direction performances. For instance, a number of studies (Wong et al., 2010; Rubley et al., 2011; Sander et al., 2013; Ozbar et al., 2014; Granacher et al., 2015; Prieske et al., 2016) already examined the effects of different resistance training programs (e.g., plyometric training) in young soccer athletes and found significant improvements in strength, power, linear and/or change-of-direction speed following training. Another possible explanation for the observed inconsistent performance gains over the season could be that body mass status (i.e., body mass index) was inappropriate. In fact, Nikolaidis (2014) argued that physical fitness is related to the body mass index of female soccer players. More precisely, this author reported larger performance output (e.g., leg muscle power) in athletes with a body mass index of ~22 kg/m^2^ compared to those athletes with lower and higher values. In the present study, our athletes' body mass index did not significantly change over the course of the season and it ranged from 20.4 to 20.7 kg/m^2^. Thus, it is postulated that the observed inconsistent performance gains in muscle power could be due to their low body mass index.

It appears that during the preparation periods, a particular focus should be laid on the promotion of physical fitness to prepare the athletes for the competition period. In accordance with this approach, our training data indicated a great percentage of sport-specific training especially during the competition periods (64–68%) and less sport-specific training during the preparation periods (48–52%). However, our detailed analyses of the single training periods showed significant improvements, both during the preparation (e.g., muscle endurance, endurance, and balance performance during PP1 [4–27%]) and the competition periods (e.g., jump performance during CP1 [19–38%]). Thus, it appears that the training stimuli during the competition periods provided sufficient overload for performance enhancements. Further, more in-depths analyses of our data revealed that physical fitness changes occurred during the first round of the season only. This might indicate either less effective training stimuli or less optimal physical and/or psychological conditioning (e.g., imbalance between stress and recovery) during the second round of season. For instance, Noon et al. (2015) found moderate-to-large decrements in perceptions of well-being (e.g., motivation, sleep quality, appetite, fatigue) and several physical fitness tests (e.g., 30 m sprint) in English elite young soccer players aged 17 ± 1 years as the soccer season progressed. They concluded that their findings are indicative of an imbalance between stress and recovery during the season; even if players participate less in training sessions. In terms of the transition period, the present findings indicate a significant performance decline in addition to the impaired anthropometric and body composition characteristics. Hence, our data indicate that a well-designed transition period (e.g., structured home training plan) is very important to prevent anthropometric, body composition as well as physical fitness deteriorations in the middle of the season and create optimal conditions for the onset of the second half of the season.

In this study, correlation coefficients between training types/match playing time and changes in physical fitness ranged between −0.541 and 0.505. Only a few studies examined associations between variations in seasonal training data, match playing time, and changes in anthropometry, body composition, and/ or physical fitness (Castagna et al., 2013; Silva et al., 2014; Los Arcos et al., 2015; Jaspers et al., 2017). For instance, Jaspers et al. (2017) conducted a systematic review and examined the relationship between training load indicators and physical fitness outcomes, injury and illness in adult elite soccer players. Our findings are in accordance with those from Jaspers et al. (2017) in as much as both studies observed positive to negative correlations between training volume and/or match playing time and long-term changes in physical fitness. As one possible explanation, Jaspers et al. (2017) suggested that a non-linear relationship (e.g., U-shaped curve) between the dose of (i.e., workload) and response to training (i.e., changes in physical fitness) may have contributed to the inconsistent findings reported in the literature. However, future research should continuously monitor training data to further our knowledge on these relationships.

Finally, it should be acknowledged that the lack of a passive control group represents a methodological limitation of this study. However, the inclusion of a passive control group (i.e., no soccer training) is impossible in an elite athletic setting because we cannot expect athletes to stop training for an entire season. Moreover, our findings on anthropometry, body composition, and components of physical fitness should be considered with caution because they might also be affected by natural variation of performance and/or maturity-related changes of our sample. Additionally, the sample size appears to be rather small. However, we would like to point out that female elite young athletes were included in the present study which is why the overall population for recruitment is highly limited in an elite athletic setting.

## Conclusion

The present study clearly showed large variations in training and performance data as well as anthropometrics and body composition in female elite young soccer players who completed the season with the national under-17 championship title. The seasonal soccer training varied with respect to the training periods and thus mainly followed the principles of training variation and specificity. In addition, body composition (i.e., lean body mass, body fat mass) varied according to the demands of the respective training periods which is indicative of biopositive changes (e.g., decreases in fat mass and increases in lean body mass) during the CP1 and bionegative changes (e.g., increases in fat mass and decreases in lean body mass) during TP. Further, anthropometry (i.e., body height) changed due to maturation over the season (T1 vs. T6). Moreover, soccer training and/or growth/maturation contributed to significant gains in a number of physical fitness outcomes (i.e., DJ performance index, Y balance performance, shuttle run performance and kicking velocity) over the soccer season. This is particularly due to adaptations during the first round of the season (i.e., PP1, CP1). It is noteworthy that other performance data did not change (i.e., SJ height, CMJ height, DJ height, 10 m-sprint, change-of-direction speed, ventral Bourban test) or even decline (i.e., maximal leg extensor strength) over the season (T1 vs. T6) which might be caused by fatigue and/or insufficient training stimuli. Thus, it is recommended that coaches and practitioners should carefully consider the training volume of jumping, sprinting, agility/change-of-direction speed and/or heavy-resistance training routines in order to develop speed-, power-, and strength-related fitness measures more efficiently throughout the soccer season in female elite young soccer players.

## Author contributions

Substantial contributions to the conception or design of the work; or the acquisition, analysis, or interpretation of data for the work: ML, NH, OP, UG. Drafting the work or revising it critically for important intellectual content: ML, NH, OP, UG. Final approval of the version to be published: ML, NH, OP, UG. Agreement to be accountable for all aspects of the work in ensuring that questions related to the accuracy or integrity of any part of the work are appropriately investigated and resolved: ML, NH, OP, UG.

### Conflict of interest statement

The authors declare that the research was conducted in the absence of any commercial or financial relationships that could be construed as a potential conflict of interest.

## Supplementary material

Figure S1 Baseline mean values and standard deviations of anthropometrics, body composition, and physical fitness measures in female young soccer players relative to the respective measures of representative data reported in the literature. The dotted line (100%) indicates the level of the outcome measures assessed in the respective age-matched reference group. Lower relative values indicate that smaller absolute values (e.g., lower t-agility test time) were assessed in female young soccer players compared to the reference group from the literature. In contrast, higher relative values indicate larger absolute values (e.g., larger squat jump height) in female young soccer players compared to the reference group.
