# Supplementary figures and images for: Effects of Soccer Training on Anthropometry, Body Composition, and Physical Fitness during a Soccer Season in Female Elite Young Athletes: A Prospective Cohort Study
Source: Front Physiol. 2017 Dec 22;8:1093. doi: 10.3389/fphys.2017.01093 (PMC5770736; doi:10.3389/fphys.2017.01093)

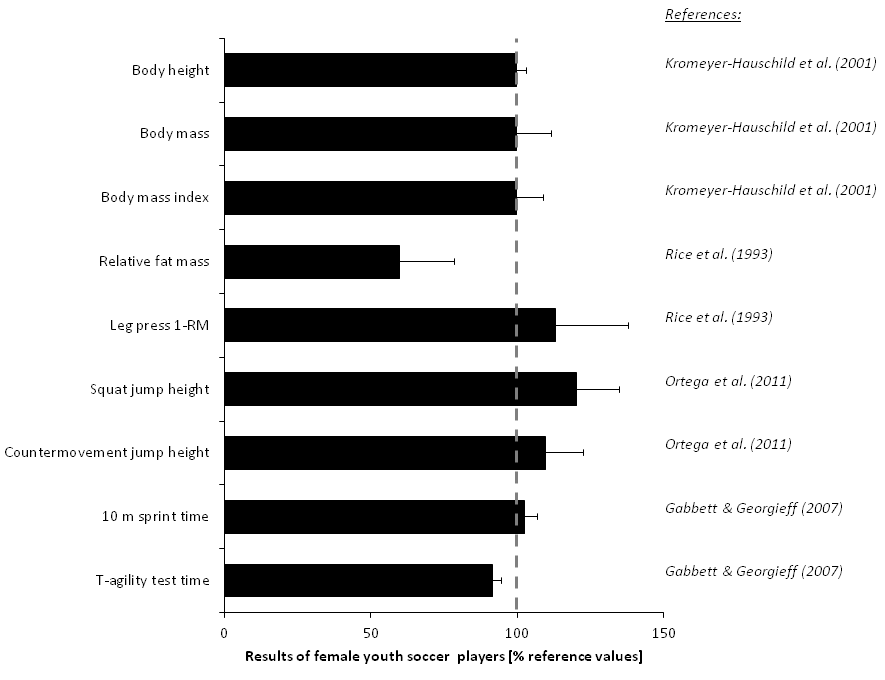

Supplement: Figure S1 — Baseline mean values and standard deviations of anthropometrics, body composition, and physical fitness measures in female young soccer players relative to the respective measures of representative data reported in the literature. The dotted line (100%) indicates the level of the outcome measures assessed in the respective age-matched reference group. Lower relative values indicate that smaller absolute values (e.g., lower t-agility test time) were assessed in female young soccer players compared to the reference group from the literature. In contrast, higher relative values indicate larger absolute values (e.g., larger squat jump height) in female young soccer players compared to the reference group. [file Image1.TIF]
